# What properties characterize the hub proteins of the protein-protein interaction network of *Saccharomyces cerevisiae?*

**DOI:** 10.1186/gb-2006-7-6-r45

**Published:** 2006-06-16

**Authors:** Diana Ekman, Sara Light, Åsa K Björklund, Arne Elofsson

**Affiliations:** 1 Stockholm Bioinformatics Center, Stockholm University, Stockholm, Sweden

## Abstract

**Background:**

Most proteins interact with only a few other proteins while a small number of proteins (hubs) have many interaction partners. Hub proteins and non-hub proteins differ in several respects; however, understanding is not complete about what properties characterize the hubs and set them apart from proteins of low connectivity. Therefore, we have investigated what differentiates hubs from non-hubs and static hubs (party hubs) from dynamic hubs (date hubs) in the protein-protein interaction network of *Saccharomyces cerevisiae*.

**Results:**

The many interactions of hub proteins can only partly be explained by bindings to similar proteins or domains. It is evident that domain repeats, which are associated with binding, are enriched in hubs. Moreover, there is an over representation of multi-domain proteins and long proteins among the hubs. In addition, there are clear differences between party hubs and date hubs. Fewer of the party hubs contain long disordered regions compared to date hubs, indicating that these regions are important for flexible binding but less so for static interactions. Furthermore, party hubs interact to a large extent with each other, supporting the idea of party hubs as the cores of highly clustered functional modules. In addition, hub proteins, and in particular party hubs, are more often ancient. Finally, the more recent paralogs of party hubs are underrepresented.

**Conclusion:**

Our results indicate that multiple and repeated domains are enriched in hub proteins and, further, that long disordered regions, which are common in date hubs, are particularly important for flexible binding.

## Abstract 2

An analysis of hubs (proteins with many interactors) and non-hubs in the *S. cerevisiae *protein interaction network shows that hub proteins are enriched with multiple and repeated domains.

## Background

Physical interactions between proteins are fundamental to most biological processes, since proteins need to interact with other proteins to accomplish their functions. Hence, knowledge about the interactions between proteins is crucial for understanding biological functions. Furthermore, the functions of many proteins are unknown and identification of the physical interactions in which these proteins participate is likely to give an indication of their function. In the past few years new technologies have facilitated high-throughput determination of protein-protein interactions. In large-scale experiments, tandem-affinity purification (TAP) followed by mass spectrometry is a common technique for identifying protein complexes [1], while the yeast two hybrid method is used for identifying individual protein-protein interactions [2-4]. Once a large subset of the interactions between proteins has been characterized, the topology of the network and its evolution can be investigated. There are approximately 16,000 to 40,000 interactions between the approximately 6,000 proteins in *Saccharomyces cerevisiae *[5,6].

The identified protein-protein interaction network (PPIN) of *S. cerevisiae *shows a power-law connectivity distribution [7]. A distribution with these characteristics indicates that a few proteins are highly connected (hubs) while most proteins in the network interact with only a few proteins. However, since the coverage of the real PPIN is low, it has been questioned whether the topology of the PPIN can currently be correctly identified [8]. Even if the exact nature of the degree-distribution of the PPIN has not been correctly determined, it is clear that some highly connected proteins are characterized by certain properties. For instance, the hubs are about three times more likely to be essential to *S. cerevisiae *compared to their non-hub counterparts [7]. It is conceivable that hub proteins could be particularly interesting drug targets, for instance in cancer research [9], where hub proteins that are highly expressed in diseased tissues may be targeted.

The hubs of the PPIN of *S. cerevisiae *have been shown to evolve slowly, which may be because larger portions of the lengths of these proteins are directly involved in their interactions [10,11]. In contrast, other studies indicate that the proposed negative correlation between evolutionary rate and connectivity is only due to a small fraction of proteins with high numbers of interactions that evolve slower than most proteins in the yeast network [12]. The difference between some of these studies seems to be due to the nature of the data sets. When complexes identified with mass spectrometry based methods are included in the analysis, the relationship between connectivity and evolutionary rate is clear [13].

Based on expression profiles it is possible to distinguish two different hub types in the PPIN of *S. cerevisiae*; static hubs (party hubs) and dynamic hubs (date hubs) [14]. The party hubs are found in static complexes where they interact with most of their partners at the same time, while the date hubs bind their interaction partners at different times and/or locations. Party hubs are thought to be the central parts of functional complexes while date hubs act as the organizing connectors between these semi-autonomous modules. Thus, date hubs appear to be more important than party hubs for the topology of the network [14]. Further, while there is no substantial difference between the proportion of essential proteins among the party and date hubs, perturbation of the latter leads to sensitization of the genome to further perturbations [14]. In addition, the phylogenetic distribution is broader for party hubs compared to date hubs [15].

Here, we seek to identify whether additional functional, evolutionary or structural properties distinguish hubs from non-hubs and date hubs from party hubs.

## Results and discussion

We used the computationally verified core data set [16] from the database of interacting proteins (DIP) [17] to build a representation of the PPIN of *S. cerevisiae*. The data set consists of 2,640 protein nodes and 6,600 interaction edges. In addition to DIP, we performed all the studies described herein on the filtered yeast interactome (FYI) data set used by Han *et al*. [14].

The connectivity (k) of a protein is defined as the number of proteins with which it interacts. To study the characteristics of the hubs in the yeast interaction network, we have divided the proteins into three groups based on their connectivities. This yields 519 highly connected proteins (hubs; k ≥ 8), 577 intermediately connected proteins (4 ≤ k ≤ 7) and 4,792 non-hubs (NH; k ≤ 3). The hubs were further classified as static party hubs (PHs) or dynamic date hubs (DHs), where party hubs are believed to interact with most of their partners at the same time while date hubs interact with their partners at different times and/or locations. The classification was based on the expression profiles of the hubs, as described by Han *et al*. [14].

**Figure 1:**
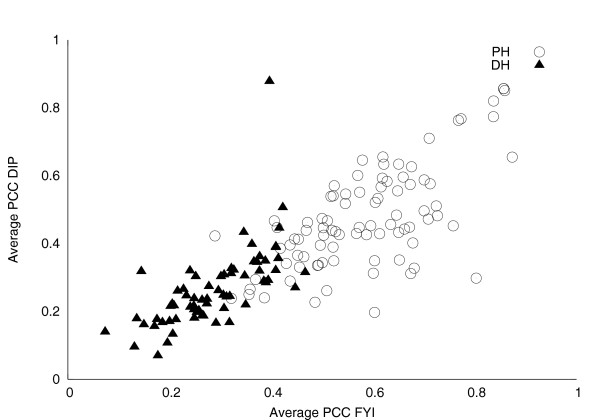
Co-expression in FYI and DIP. Average PCCs of the co-expressions of party hubs (PHs) and date hubs (DHs) and their interaction partners were calculated for the FYI-defined PH and DH. Average PCCs calculated for the interaction partners in the FYI network (*x *axis) correlate (CC = 0.8) with the average PCCs calculated within the DIP network (*y *axis). The values in the DIP network are on average lower.

**Figure 2:**
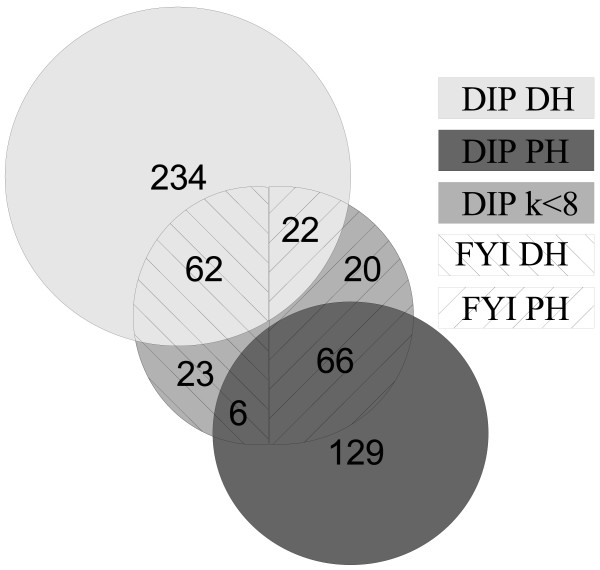
Hub assignment. The overlap between date hubs (DHs) and party hubs (PHs) in the two data sets; DIP and FYI. In FYI there are 108 PHs and 91 DHs (middle circle), of which 23 DHs and 20 PHs have connectivities below the hub threshold (k < 8) in DIP. Most of the FYI PHs (66) were confirmed as PHs in the DIP set, while 22 fell below the PCC cutoff (see Materials and methods). Furthermore, while most of the FYI DHs retained their DH status using DIP, a small fraction of the FYI DHs (6) were classified as PHs. Finally, 234 and 129 previously unclassified hubs were assigned as DHs and PHs in DIP.

Naturally, the hub sets in DIP and FYI do not overlap perfectly. There are hubs in DIP that cannot be classified as hubs in FYI due to low connectivities in that data set, and conversely, FYI hubs whose connectivities fall under the hub threshold in DIP. Furthermore, the coexpression analysis gives slightly different party hub and date hub classifications as the Pearson correlation coefficient (PCC) values in the DIP set on average are lower than in the FYI set (Figure 1). After adjustment of the cutoffs, most of the FYI party hubs also qualify as party hubs in the DIP network and the FYI date hubs as DIP date hubs (Figure 2). The resulting number of proteins in each category in the respective data sets and their average connectivities can be found in Table 1. Unless otherwise stated, the results derived from the two data sets were qualitatively similar. It should be noted, however, that the number of interactions in the DIP set is substantially larger, resulting in larger separation between the connectivity groups.

**Figure 3:**
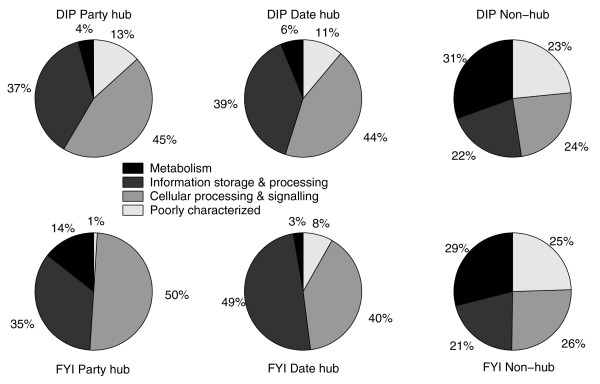
Functional classification of party hubs, date hubs and non-hubs. The functional classification was performed using KOG [19]. This classification consists of four main functional groups: metabolism; information storage and processing; cellular processes and signaling; and poorly characterized. Unnamed proteins have been excluded, although this is fairly common among the non-hub proteins.

The reason why some proteins interact with a multitude of proteins and others interact with only a few is not well understood. Clearly, the connectivity of a protein is related to its function [18]. We found, using KOG [19] functional classification, that high connectivity is often associated with proteins involved in 'Information storage and processing' (transcription in particular) and 'Cellular processes and signaling'. Among the non-hubs, on the other hand, there are many proteins that participate in metabolism (Figure 3), and as expected, proteins with poorly characterized functions frequently have few or no interactors. However, it is important to bear in mind that there are numerous possible sources of bias in the PPIN data that may affect these results. For instance, since conserved proteins may be particularly interesting for scientific studies, there could be some experimental bias for these interactions while there is a possible bias against yeast-specific interactions [20] and interactions involving membrane proteins.

### The phylogenetic distribution of hub proteins

A recent study showed that party hubs are found in more eukaryotic species than date hubs [15]. Here, we analyze the phylogenetic distribution, as an estimate of age, of the proteins belonging to the different connectivity groups. Our study shows that a larger fraction of the hub proteins, and particularly party hubs, have eukaryotic orthologs compared to the non-hubs (Table 2). Furthermore, party hubs more often have orthologs in prokaryotes than do date hubs.

**Figure 4:**
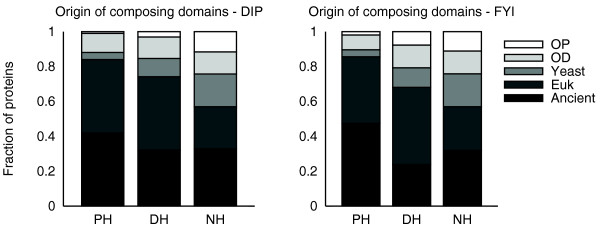
Protein age. The age of a protein is here estimated from the age of its domains. Domains may be found in: eukaryotes and prokaryotes (Ancient); eukaryotes (Euk); or yeast. Domains and proteins that lack homologs are called orphan domains (ODs) and orphan proteins (OPs). The age of a single domain protein is equal to the age of its composing domain, whereas each domain family represented in a multi-domain protein contributes equally to its age classification. Furthermore, each protein contributes equally to the age of its connectivity group. Hence, a two-domain protein may be half ancient and half eukaryotic. The figure shows fractions of proteins, that is, party hubs (PHs), date hubs (DHs) and non-hubs (NHs) in each age class in DIP and FYI.

The domain contents of the proteins may provide further clues about protein age [21]. Therefore, we assigned Pfam [22] domains to all proteins and studied the phylogenetic distribution of the domains. The domains were classified as ancient (found in eukaryotes and prokaryotes), eukaryote specific, yeast specific or orphan (no homologs) (Figure 4). Consistent with the results from the ortholog analysis, the fraction of orphan and yeast specific domains in hubs is smaller than for non-hubs. There are further differences between the hub types; the party hubs have a higher fraction of ancient domains and few yeast specific domains compared to date hubs.

In conclusion, the phylogenetic distribution of orthologs and the domain content imply that hubs, particularly party hubs, often are older than non-hubs. The non-hub group seems to be a mixture of proteins of recent origin and ancient proteins, whose low connectivity is probably related to the large fraction of proteins with metabolic functions. These results are consistent with the finding that connectivity is related to protein age, although the oldest proteins are not necessarily the most highly connected [18].

### Duplicability of hub proteins

The protein-protein interaction network is susceptible to targeted attacks on the hubs of the network [7,23]. Since hub proteins are pivotal for the robustness of the protein-protein network, it is conceivable that the *S. cerevisiae *genome may contain more genetically redundant duplicates of the hubs compared to other proteins. On the other hand, gene duplications may cause an imbalance in the concentration of the components of protein-protein complexes that might be deleterious [24,25]. The first mechanism predicts that the hubs should have a higher fraction of paralogs than other proteins. In contrast, the latter mechanism, which is sometimes referred to as dosage sensitivity, predicts the opposite.

**Figure 5:**
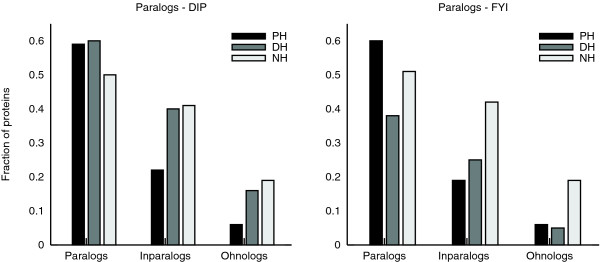
Paralogs. Fraction of proteins, that is, party hubs (PHs), date hubs (DHs) and non-hubs (NHs), that have paralogs, inparalogs (i.e. paralogs that have been duplicated after the split between *S. cerevisiae *and *S. pombe*) and ohnologs (paralogs resulting from the whole genome duplication). In DIP, the fraction of party hub inparalogs is small, approximately 0.2 compared to approximately 0.4 for the other connectivity groups (*P *value <10^-5^), and so is the fraction of ohnologs for party hubs compared to the other groups (*P *value <10^-5^). The results in the FYI data set are similar, although the fraction of date hub paralogs is smaller than in the DIP data set.

We found that the fraction of hubs that have paralogs, that is, duplicated proteins, is only slightly higher than for non-hubs in the DIP set, while no significant difference is noted in the FYI set. The small difference is in agreement with a recent study [26]. In addition, we investigated the distribution of recent paralogs between connectivity groups. *S. cerevisiae *specific paralogs from the orthologous groups of KOG are likely to be recent paralogs that evolved after the split between *S. cerevisiae *and *Schizosaccharomyces pombe*, which occurred 330 to 420 million years ago. We here refer to these paralogs as inparalogs [27]. Our results show that fewer party hubs have inparalogs than other proteins (Figure 5), which suggests that dosage sensitivity may be more important for the recent paralogs of party hubs than for the older paralogs.

The ancestor of *S. cerevisiae *experienced a whole genome duplication (WGD) event roughly 100 million years ago after the divergence of *Saccharomyces *from *Kluyveromyces *[28]. Therefore, paralogous pairs of proteins pertaining to the WGD event comprise a subset of the inparalog group. Single gene duplications may result in a concentration imbalance of the components of protein-protein complexes [24,25]. A similar concentration imbalance does not arise immediately subsequent to a WGD event but could occur later if the duplicate genes are lost independently. Therefore, it might be expected that the paralogs originating from this event, the ohnologs [29], could be retained in the genome, as in the case of the ribosomal genes [25]. There is a total of 551 pairs of retained ohnologs. Interestingly, we found that the fraction of party hub proteins that were retained is somewhat lower than the corresponding fractions for date hubs and non-hub proteins (Figure 5). This result suggests that the balanced dosage of the complex components after the WGD event was insufficient to promote party hub retention.

After the duplication, both copies may retain the same set of interaction partners, or interactions could be lost and new partners gained. In accordance with a previous study [30], there is only a negligible correlation in connectivity (Cc = 0.05) between paralogs. Here, we studied proteins with one single paralog only, since the relationship between proteins in larger families is harder to establish. However, the paralogs of hubs are more likely to be hubs themselves (45%) compared to non-hubs (4%), which supports the redundancy theory. Naturally, there is, in some cases, a sizable overlap between the interactions of hubs and their paralogs. It is possible that the paralogs of hub proteins provide distributed robustness, which is likely to be important for mutational robustness [31], to the PPIN, by sharing some of the functionality of the hubs. Alternatively, these are pairs of proteins from recent duplications where overlapping interactions have not yet been lost.

In conclusion, we observe a smaller fraction of recent party hub duplicates in *S. cerevisiae *compared to the fraction of recent duplicates for other proteins. Further studies are needed to determine the cause of this observation but it may be the result of a relative increase in dosage sensitivity for party hubs.

### The impact of domain content, repeats and disordered regions on connectivity

One reason for the higher complexity of eukaryotes compared to prokaryotes is the increased number of domain combinations found in eukaryotes, where, for example, binding domains have been added to existing catalytic proteins [21,32]. The idea that multi-domain proteins can bind many different proteins is intuitively appealing. Indeed, a large fraction of the proteins in the network contain multiple domains. Moreover, our results show that the proportion of multi-domain proteins in hubs is larger than the corresponding fraction in the, on average shorter, non-hubs (*P *value <10^-5^; see Materials and methods; Table 1).

**Figure 6:**
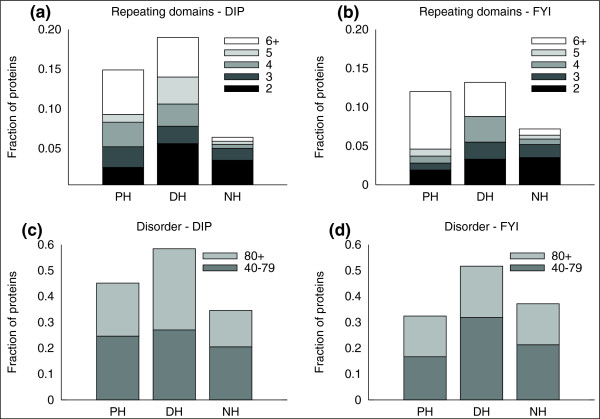
Repeating domains and disorder. Results are shown for party hubs (PHs), date hubs (DHs) and non-hubs (NHs). Repeating domains in **(a) **DIP and **(b) **FYI. A domain repeat is defined as two or more adjacent domains from the same family. Fractions of proteins with domain repeats containing 2, 3, 4, 5 or 6 or more domains are displayed. Fractions of proteins in **(c) **DIP and **(d) **FYI with disordered regions of lengths 40 to 79 residues and 80 or more residues are shown. Although 40 residues is a common cut-off for disordered regions, it is somewhat arbitrary and, therefore, 80 residues was added as an alternative cut-off.

Many repeating domains have binding functions. The WD40 repeat, for example, functions in the formation of a multi-protein complex in transcription regulation and cell-cycle control [33]. Therefore, it may be expected that proteins with domain repeats are associated with high connectivities. Consistently, hub proteins contain an increased fraction of proteins that contain domain repeats compared to non-hubs (*P *value <10^-5^; Figure 6). The difference persists after exclusion of the two most common repeating domains in this data set, WD40 and HEAT, and is hence not attributed to a single domain family. In addition, we found a similar difference between hubs and non-hubs in the interaction network of *Drosophila melanogaster *(data not shown). The results do not seem to be caused by elevated fractions of repeat proteins in certain highly connected functional classes, since they persist in all four classes (data not shown). While the intermediately connected (IC) proteins display characteristics that fall in-between those of the hub and non-hub groups, it is noteworthy that the domain repeats in the IC group are nearly as scarce as among the non-hubs.

Disordered regions, that is, regions that lack a clear structure, have been suggested to be important for flexible or rapidly reversible binding, but may also serve as linkers between domains [34-36]. These regions are found extensively in proteins pertaining to functional classes associated with high connectivities, such as transcription, cell cycle control and signaling [18,34]. In contrast, proteins involved in metabolism rarely contain disorder [37]. The binding flexibility may result in higher connectivities for proteins containing such regions [38]. Indeed, we found that hubs contain long disordered regions (≥ 40 residues) more often than non-hub proteins (Figure 6), and the difference is larger for longer disordered regions (≥ 80 residues). Interestingly, however, it is only among the date hubs that long disordered regions are significantly enriched (*P *value <10^-5^), which is even more pronounced in the FYI data set (Figure 6d).

It is possible that long disordered regions are predicted more frequently in longer proteins. To test if the over representation of long disordered regions in date hubs was in fact an artifact of the longer average length of the proteins in this group, we created a subset consisting of 3,218 non-hubs with a similar length distribution to that of the hubs. The fraction of proteins with long disordered regions increased slightly (to 41%) but was still significantly lower than the fraction in date hubs. Therefore, disorder seems to be a genuine characteristic of date hubs. Naturally, many short proteins were removed, and the fraction of multi-domain proteins increased in the length-normalized subset of non-hubs so that the fraction become similar to the hub set. In contrast, the lower fraction of proteins with repeated domains among non-hubs remained.

In conclusion, hubs are more often multi-domain proteins compared to non-hubs and they frequently contain repeated domains. Furthermore, date hubs contain more disordered regions than party hubs, which suggests that disordered regions are particularly important for the flexible binding of date hubs.

### The interaction partners of hub proteins

Hubs, by definition, bind to a large number of proteins. According to a previous study, proteins with high connectivities bind to proteins of low connectivity [39], and they often bind to proteins that originate from the same period in evolution [40]. In addition, proteins that interact often belong to the same functional category [20]. Clearly, the nature of the interactions in which the party hubs are involved may be different from that of the date hub interactions, since, for example, the latter interactions are more likely to be transient. In the previous section we showed that date hubs have a larger proportion of long disordered regions compared to party hubs, which indicates that the disordered regions may be important for flexible binding. To further elucidate the difference between the interaction properties of party hubs and date hubs, we have studied their respective clustering coefficients and interaction partners.

**Figure 7:**
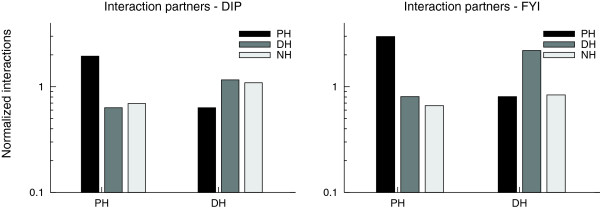
Interaction partners for party hubs (PHs) and date hubs (DHs). The displayed values are normalized fractions of the interactions (Normalized Interactions) that involve party hubs, date hubs or non-hubs for PH and DH, respectively. The values are normalized against the number of interactions that involve the respective protein types in the network. Hence, Normalized Interactions >1 signify that the given interaction pair (for example, PH-PH) is overrepresented compared to other interactions with PH, which is seen both in DIP and FYI.

**Figure 8:**
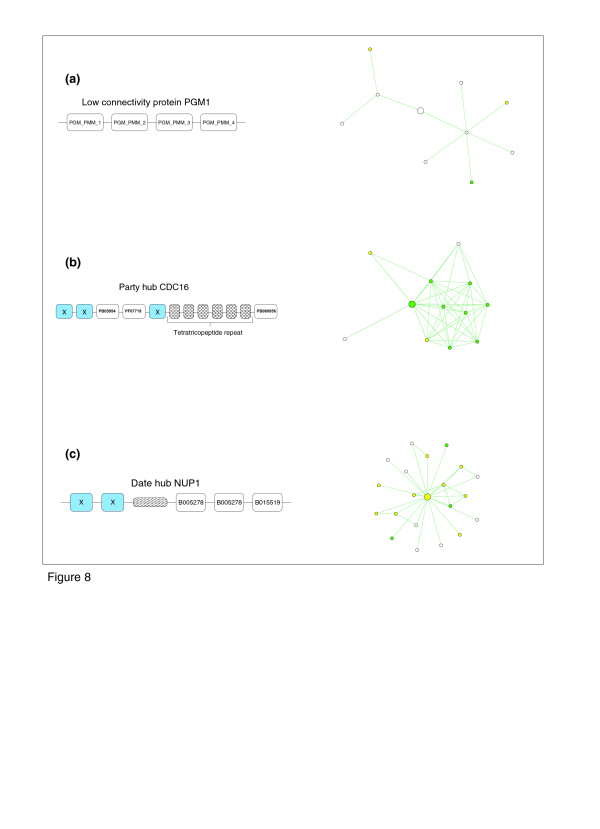
Neighbors of proteins of low connectivity (white nodes), party hubs (green nodes) and date hubs (yellow nodes); an example. a) Non-hub protein PGM1 (YKL127W, large node) is the metabolic enzyme phosphoglucomutase, which consists of four well characterized domains associated with phosphoglucomutase activity. PGM1 is only connected to two other proteins, which are not hubs. b) Party hub protein CDC16 (YKL022C, large node) is an essential protein and is part of the anaphase-promoting complex (APC). It contains six tetratricopeptide domains, one additional Pfam-A domain, two Pfam-B domains and three orphan domains (blue rectangles). CDC16 interacts with party hubs, date hubs as well as two IC and NH proteins. c) Date hub protein NUP1 (YOR098C, large node) is a nuclear pore complex protein of diverse function which contains three Pfam-B domains, two orphan domains and one long disordered region (dashed). It interacts with other date hubs, party hubs and several non hub proteins. The network figures were drawn using BioLayout[52].

It is notable that party hubs often interact with each other (Figure 7). Consistently, party hubs have neighbors that often interact, as seen by the higher clustering coefficient for party hubs (0.27) than for date hubs (0.18) (*P *value <10^-5^; Figure 8). Our data suggest that the previously observed small number of connections between highly connected proteins [39] is restricted to a limited number of interactions between date hubs and party hubs, which might translate into a small number of connection paths between the functional modules represented by the party hubs.

Further, we wanted to investigate how specialized the hubs are in their binding. In other words, are these highly connected proteins hubs because they interact with many similar proteins, or because they are able to interact with many different partners with diverse domain compositions? If hubs gained interactions through duplication of their neighbors, many neighbors would be paralogs. This has been found in some complexes, which consist of paralogous sequences [41], for example, the Septin ring. However, interactions are often lost by one of the paralogs soon after duplication [30]. Consistently, in our data set there is an average of approximately 1.2 sequences from each paralogous family in the hub-interacting proteins, that is, only a small fraction of the interactions can be explained by interactions with paralogs. A looser definition of homology is the sharing of a domain family. A domain that is recurring in all neighbor proteins could also provide a necessary binding site; however, binding may sometimes be mediated by short linear motifs [42]. Here, we refer to the domain shared by the largest number of the neighboring proteins as the most frequently shared domain (MFSD).

There are examples of proteins that interact only with proteins containing the MFSD and other flexible proteins that interact with more than 30 different proteins where only a few of the interactors share a domain (Additional file 2). Some domain families are more likely to be shared by a large number of the neighbors. The most frequent MFSDs are Pkinase and WD40, which are the MFSDs for more than 50 hubs each. Certainly, there is a recurrence of domain families in the interacting proteins of most hubs; on average, however, only one fourth of the interacting proteins share the MFSD, both in party hubs and date hubs, which is still more than expected in a random network (0.11, *P *value <<10^-5^). Furthermore, in as many as 23% of the hubs, the MFSD in the interactors is shared with the hub, a feature almost twice as frequent in party hubs as in date hubs. Such same-domain-interactions (SDIs) are found between proteins containing, for example, Pkinase, LSM, proteasome and AAA domains, and, among all the interaction pairs in the PPIN, 7.6% of the interactions are SDIs, which is more than expected in a randomized network (1.2%, *P *value <10^-5^). Thus, the party hubs often contain the domains that are most common among their interaction partners. This is, at least partly, due to the fact that some complexes consist of several paralogous sequences.

However, our results indicate that hubs do not interact particularly often with paralogous groups of proteins. Neither can recurrence of domains in interaction partners explain much of the interactions in the network. Furthermore, we noted that multi-domain hub proteins have somewhat more diverse binding partners than single domain hubs. The partner flexibility also seems to be higher in proteins with disordered regions or domain repeats (data not shown). In conclusion, the high connectivity of hub proteins in the *S. cerevisiae *PPIN can, to some extent, be explained by disorder, domain repeats, several binding sites, interactions with and between homologous proteins as well as proteins consisting of domains associated with many diverse binding partners, such as kinases.

## Conclusion

We found that the duplicability of hub proteins is similar to that of other proteins. However, very few static hub (party hub) paralogs originate from relatively recent duplications. We hypothesize that the number of retained party hub duplicates has decreased relative to the duplicates of non-hubs during the evolution of *S. cerevisiae*. Although there may be other explanations, it is possible that the dosage sensitivity of party hubs has increased in comparison to other proteins through evolution.

An important question is what leads to the high connectivity of hub proteins? Perhaps surprisingly, our findings show that domain recurrence among hub interaction partners can only explain some of the interactions in the network and, furthermore, hubs do not interact particularly often with paralogous groups of proteins. It is quite likely that the interaction data sets contain at least some indirect interactions, that is, interactions mediated through a third protein. In particular, interaction data sets derived from TAP data could be rich in such interactions. Nevertheless, we found that some properties are common among the hub proteins of the *S. cerevisiae *protein-protein interaction network. There is an enrichment of multi-domain proteins among the hub proteins compared to non-hub proteins, and they are, on average, longer. Moreover, repeated domains are clearly over-represented in hub proteins. The presence of repeated domains and multiple domains in hubs may partly explain their high connectivities.

Finally, there are properties that differentiate the party hubs from the dynamic hubs (date hubs). For instance, the party hubs self-interact to a greater extent than date hubs. In addition, party hubs interact with proteins with which they share domains more often than date hubs, whereas date hubs contain more long disordered regions. Our findings suggest that while repeats and multiple domains promote protein-protein interactions in general, disordered regions are of particular importance for the flexible interactions of date hubs.

## Materials and methods

### The protein-protein interaction network

The PPIN was built using the 'core' data set from the DIP [16,17] downloaded in March 2005. A second PPI data set was also used, the FYI from Han *et al*., which contains 1,379 proteins with 2,493 interactions [14]. The PPI data for *D. melanogaster *was downloaded from the DIP in January 2005.

### Protein classification in the network

The connectivity (k) of a protein node is defined as the number of proteins it is connected to, including possible self-interactions. The proteins were grouped according to their connectivities in the core interaction network. Hubs are defined in DIP as proteins with eight or more interactions while proteins with less than four interactions are named non-hubs and the rest are intermediately connected. For simplicity, the results for the latter group are not described here. Unless otherwise stated, the results for this group are, as expected, in-between those of the hub and non-hub groups. The number of proteins and the average connectivities for the respective groups are found in Table 1. Hubs in FYI are proteins with k ≥ 6 [14], whereas non-hubs have k ≤ 1. We chose to use different cutoffs for non-hubs in order to include similar numbers of proteins in this group in both data sets.

### Defining party hubs and date hubs

The annotation of hubs as party (PH) and date (DH) hubs was collected from Han *et al*. [14] for the FYI data set. The same approach was adapted from Han *et al*. [14] to define party and date hubs in the DIP data set. Co-expression profiles from five different conditions (stress response [43], cell cycle [44], pheromone treatment [45], sporulation [46] and unfolded protein responses [47]) were normalized with Z score normalization using the original log_2 _fold change values. The average PCC between each hub and its interaction partners was calculated for the five conditions and for the combined set of all conditions. Party hubs are defined as proteins that show high average PCC with their interaction partners. In the FYI set there was a bimodal distribution of the average PCC values, which was used to distinguish party hubs from date hubs. However, we found no clear bimodal distribution in the DIP set and, in addition, the average PCC values were generally lower in the DIP set than in the FYI set (Figure 1). Therefore, we used lower thresholds for separating date and party hubs in the DIP set. To maximize the overlap between the hub classifications in FYI and DIP, the threshold was set to 0.4 for all conditions, except for the combined set and stress response (0.35) as well as cell cycle (0.3). Clearly, the choice of thresholds is somewhat arbitrary. However, increasing or decreasing the threshold by 0.1 has only minor effects on the results.

The difference between the hub classifications derived from the DIP and FYI datasets is shown in Figure 2. A number of ribosomal hub proteins were excluded from the FYI classification [14]. As the connectivity of these proteins in the DIP set was below the hub cutoff, this was not necessary here.

### Clustering coefficient

For a node N with n neighbors there are ((*n*) × (*n *- 1))/2 possible undirected edges between the n neighbor nodes. The clustering coefficient is the actual number of edges between the neighbors n of N divided by the number of possible edges between the nodes.

### Domain assignment

Domains from Pfam-A [22] were assigned to each sequence in the yeast genome with HMMER-2.0 [48], using a cutoff of 0.1. Repeating domains, defined as two adjacent domains from the same family, are often not recognized at this threshold; therefore, the threshold for including an additional domain from the same family was set to 10. Pfam-B domains were then assigned to sequences and regions lacking Pfam-A domains. The number of domains in proteins was determined according to a procedure presented elsewhere [21] as the sum of the number of Pfam-A and Pfam-B domains and unassigned regions longer than 100 residues (orphan domains). The yeast protein sequences were collected from the *Saccharomyces *Genome Database [49]. Only verified open reading frames were included. Finally, disorder was predicted with Disopred2 [34] at a 5% expected rate of false positives.

### Protein age

Protein age was estimated from the domain contents. Domains were assigned to 21 species, 7 from each kingdom (for species see Ekman *et al*. [21]). The domains were then grouped into those present in: eukaryotes and prokaryotes; eukaryotes only; *S. cerevisiae *and/or *S. pombe *(yeast); and no species (orphans). Proteins with no domain assignments were considered orphan proteins. To avoid bias toward repeating domains, each domain family represented in the protein contributed equally to the age class of the protein, that is, repeating domains were only counted once. Furthermore, each protein lends an equal contribution to the age of the connectivity group, irrespective of domain number. Hence, a five domain protein consisting of four ancient domains A and one eukaryotic domain B is half ancient and half eukaryotic.

### Orthologs, paralogs and functional annotation

Clusters of orthologous groups (COG) [50] and KOG [19], were used to define orthologs in prokaryotes and eukaryotes, respectively. Functional categories given by KOG were used as the functional classification of the proteins. KOG, complemented by two-species groups (TWOGs) and lineage-specific extensions (LSEs), was also used to find inparalogs [27]. All *S. cerevisiae *sequences in the same KOG, TWOG or LSE were considered inparalogs unless they had completely different domain architectures. We retrieved 551 pairs of paralogs that originate from the whole genome duplication of *S. cerevisiae *(ohnologs) from the Yeast Gene Order Browser [29] and these were also included in the inparalog set. Paralogous sequences were retrieved using a Blastp [51] all-against-all search. Paralogs were defined as sequences with an e-value below 10^-5 ^and an alignment covering more than 40% of the longest of the compared sequences. Proteins of at least two domains and identical Pfam-A or Pfam-A+B domain architectures were also considered paralogous if their lengths did not differ by more than 30% of the longer sequence. The fractions of proteins with paralogs were calculated as the number of proteins that have paralogs, that is, that are not singletons, divided by the number of proteins. Hence, if a hub is paralogous to a protein that is also a hub, both of them will be counted as proteins with paralogs.

### Statistical tests

*Z* scores were calculated in the following manner to estimate the statistical significance of our results. For example, to determine the significance of the distribution of inparalogs between party hubs and other proteins, the connectivity classes were randomized and the number of inparalogs in the two classes was calculated. This process was iterated 10,000 times and the average and standard deviation from the randomizations were used to calculate the *Z* score:

*Z *= ( - )/*stdev*(*r*)

where *n *is the real number of inparalogs in a connectivity class k and *r *is the result from the randomized network. In a similar manner, the *Z *scores for the distribution of ohnologs, clustering coefficients, proteins with repeats and proteins containing disorder were calculated.

In the case of the *Z* score calculation for the MFSD, the connections in the protein interaction network were shuffled and the domain compositions were retained. The result from the real network was compared to the result from the randomized network by calculating the *Z* score as above where  is the average proportion of proteins with the most common domain in the real network for a certain connectivity class k and  is the same number for the randomized networks. In a similar manner, the Z scores for the same-domain-interactions were calculated for the party hub and date hub groups. The *P *value was then derived from the *Z* score assuming a normal distribution.

## Additional data files

The following additional data are available with the online version of this paper. Additional file 1 lists all proteins and their classifications (PH/DH/NH). Additional file 2 is a table of all date and party hubs together with information about them (for example, connectivity, average PCC of co-expression, domain assignments, and disorder).

## Supplementary Material

Additional Data File 1 All proteins and their classifications (PH/DH/NH) All proteins and their classifications (PH/DH/NH) Click here for file

Additional Data File 2 All date and party hubs together with information about them (for example, connectivity, average PCC of co-expression, domain assignments, and disorder) Click here for file

## Figures and Tables

**Table 1:** General properties

	No. seq	<k>	Length	MD (%)
DIP				
Party hubs	201	13.4	581 ± 28	71
Date hubs	318	14.4	632 ± 27	70
Non-hubs (k ≤ 3)	4792	-	473 ± 5	60
FYI				
Party hubs	108	8.4	558 ± 39	76
Date hubs	91	10.2	576 ± 55	64
Non-hubs (k ≤ 1)	5045	-	492 ± 5	61

The proteins have been divided into party hubs, date hubs and non-hubs. The table shows the number of sequences in each group (No. seq), their average connectivity (<k>), average length with standard error and percentages of proteins with multiple domains (MD).

**Table 2:** Orthologs

	Euk ortho (%)	All species (%)	Prok ortho (%)
DIP			
Party hubs	95	61	61
Date hubs	88	53	49
Non-hubs	61	25	44
FYI			
Party hubs	96	75	61
Date hubs	78	46	31
Non-hubs	62	26	44

The proteins have been divided into party hubs, date hubs and non-hub proteins. The table shows the fraction of proteins in each group that has orthologs in other eukaryotes (Euk ortho), how many of these have orthologs in all seven eukaryotes (All species) and the fraction with orthologs in prokaryotes (Prok ortho), according to KOG [19] and COG [50].
