# Supplementary material for: What properties characterize the hub proteins of the protein-protein interaction network of Saccharomyces cerevisiae?
Source: Genome Biol. 2006 Jun 16;7(6):R45. doi: 10.1186/gb-2006-7-6-r45 (PMC1779539; doi:10.1186/gb-2006-7-6-r45)
Supplement: Additional Data File 2 — All date and party hubs together with information about them (for example, connectivity, average PCC of co-expression, domain assignments, and disorder) [file gb-2006-7-6-r45-S2.pdf]

DH	k	aPCC	Doms	Domains	Rep.doms	Diso	Int.dom	Func.
YBR160W	111	0.27	1	PF00069	No	5	17 (PF00069)	R
YNL189W	61	0.26	9	PF01749, PF00514	8 (PF00514)	37	4 (PF00076)	U
YBR109C	51	0.18	4	PF00036	4 (PF00036)	5	4 (PF00063)	T
YDR388W	50	0.25	3	PF00018, PF03114	No	160	5 (PF00018)	U
YBR009C	42	0.31	1	PF00125	No	28	3 (PF00125)	B
YMR308C	40	0.29	11	PF02985	7 (PF02985)	14	5 (PF00227)	YU
YJR022W	37	0.17	1	PF01423	No	4	8 (PF01423)	A
YIL035C	36	0.30	1	PF00069	No	30	3 (PF00125 PF00270 +)	TDK
YDR328C	36	0.10	2	PF03931, PF01466	No	16	10 (PF00646)	O
YOR181W	35	0.33	3	PF04554, PF02205, PF00568	No	518	10 (PF00018)	TZ
YGR218W	34	0.26	9	PF03810	No	22	3 (PF04096)	YU
YHR030C	33	0.23	2	PF00069	No	34	6 (PF00069)	T
YGL137W	32	0.33	7	PF04053, PF00400	6 (PF00400)	23	3 (PF00118 PF00400 +)	U
YER148W	32	0.20	2	PF00352	2 (PF00352)	20	2 (PF00382 PF04153 +)	K
YEL037C	31	0.23	4	PF00627, PF00240	2 (PF00627)	72	5 (PF00004)	L
YPL204W	30	0.19	3	PF00069	No	62	2 (PF00149 PF04499 +)	T
YDL140C	29	0.25	20	PF05001, PF04998, PF00623	14 (PF05001)	290	2 (PF01096)	K
YGR040W	29	0.13	1	PF00069	No	25	2 (PF00749 PF00083)	T
YDR192C	27	0.08	2	PfamB/OP	No	371	4 (PF03810)	YU
YML064C	27	0.18	1	PF00071	No	59	3 (PF00107)	R
YHR135C	27	0.18	3	PF00069	No	68	6 (PF00069)	T
YDL188C	27	0.18	2	PF00149	No	64	13 (PF00227)	T
YDR167W	26	0.16	1	PF03540	No	54	2 (PF07524 PF00169 +)	K
YLR147C	26	0.26	1	PF01423	No	23	11 (PF01423)	A
YER133W	25	0.17	1	PF00149	No	18	2 (PF03370 PF00400 +)	TR
YOL004W	24	0.22	13	PF02671	3 (PF02671)	223	3 (PF00628 PF00172)	B
YMR125W	24	0.21	3	PF02854	No	32	7 (PF00076)	A

YLR347C	24	0.27	10	PF02985, PF03810	6 (PF02985)	11	3 (PF04096)	YU
YML109W	24	0.28	1	PfamB/OP	No	59	3 (PF00786 PF00169)	X
YIL061C	23	0.16	3	PF00076	No	118	3 (PF00270)	A
YDR477W	23	0.29	4	PF00069	No	191	4 (PF00069)	T
YBR198C	23	0.33	9	PF04494, PF00400	6 (PF00400)	149	4 (PF00118)	K
YJR091C	22	0.16	13	PF00806, PF00076	5 (PF00806)	155	5 (PF00069)	R
YCR093W	22	0.13	12	PF04054	No	29	3 (PF04153 PF00400)	K
YHR165C	22	0.24	15	PF01398	No	135	3 (PF01423)	A
YDR448W	22	0.19	4	PF00569, PF04433, PF00249	No	59	2 (PF00439)	B
YLL039C	22	0.23	5	PF00240	5 (PF00240)	2	3 (PF00627 PF00069)	OR
YOL051W	21	0.16	1	PF05397	No	129	1 (PF05000)	
YGL112C	21	0.26	4	PF07571, PF02969	No	48	2 (PF00439 PF00133)	K
YGL172W	21	0.23	4	PfamB/OP	No	211	4 (PF03810)	YU
YIL142W	21	0.23	1	PF00118	No	17	15 (PF00400)	O
YPL153C	21	0.24	5	PF00498, PF00069	No	104	1 (PF00056)	D
YLR438C-A	21	0.01	1	PF01423	No	11	8 (PF01423)	
YPL031C	20	0.11	1	PF00069	No	4	8 (PF00134)	R
YER022W	20	0.22	4	PfamB/OP	No	51	3 (PF00170)	X
YMR213W	20	0.20	5	PF00249	2 (PF00249)	59	3 (PF00270 PF00271 +)	AD
YER095W	20	0.29	5	PF00633	No	45	2 (PF00004)	L
YER165W	20	0.32	5	PF00076, PF00658	4 (PF00076)	103	2 (PF02854 PF04096)	AJ
YOR098C	20	0.29	9	PfamB/OP	No	767	5 (PF03810)	YU
YPL240C	19	0.34	2	PF00183, PF02518	No	35	5 (PF00515)	O
YGL061C	19	0.21	2	PfamB/OP	No	64	1 (PF01920)	
YJL041W	19	0.32	6	PF05064	No	638	2 (PF00636)	YU
YLR452C	19	0.17	3	PF00615, PF00610	No	79	2 (PF05739 PF00069)	
YGR274C	19	0.39	7	PF00098	No	47	2 (PF04153 PF04065)	K
YDL145C	19	0.31	8	PF06957, PF04053, PF00400	6 (PF00400)	23	2 (PF01217 PF01602)	U

YDR176W	19	0.21	3	PfamB/OP	No	70	2 (PF00172 PF04082)	B
YPR086W	19	0.18	3	PF00382	2 (PF00382)	30	2 (PF00096 PF01399 +)	K
YNL147W	19	0.29	1	PF01423	No	20	8 (PF01423)	A
YFL017W-A	19	0.09	1	PF01423	No	6	2 (PF01423 PF00400)	
YDR099W	19	0.39	1	PF00244	No	19	2 (PF00786 PF01204 +)	O
YGL120C	18	0.20	4	PF07717, PF00270, PF00271, PF04408	No	83	4 (PF00400)	A
YGR091W	18	0.26	3	PF01798	No	46	6 (PF01423)	A
YDR212W	18	0.26	1	PF00118	No	24	11 (PF00400)	O
YLR115W	18	0.27	8	PfamB/OP	No	30	2 (PF00400)	A
YOL135C	18	0.22	1	PF05983	No	28	1 (PF07954)	K
YBL026W	18	0.24	1	PF01423	No	11	9 (PF01423)	A
YDR188W	18	0.19	1	PF00118	No	17	15 (PF00400)	O
YJL138C	18	0.31	2	PF00270, PF00271	No	13	2 (PF02854 PF00271 +)	J
YLR268W	18	0.33	3	PF00957	No	21	3 (PF05739)	U
YDR170C	17	0.20	11	PF01369	No	89	2 (PF04810 PF00626 +)	U
YNL330C	17	0.28	2	PF00850	No	38	3 (PF00628)	B
YBR081C	17	0.29	12	PF07524, PF00439	No	165	1 (PF00096)	BK
YPL042C	17	0.20	1	PF00069	No	152	2 (PF00172 PF00069)	K
YPR182W	17	0.17	1	PF01423	No	15	3 (PF01423)	A
YGR013W	17	0.27	4	PfamB/OP	No	49	5 (PF01423)	A
YIL004C	17	0.22	1	PF05739	No	71	2 (PF00025 PF04810 +)	U
YJL124C	17	0.34	1	PF01423	No	33	7 (PF01423)	A
YBR055C	17	0.21	9	PF06424	No	36	4 (PF01423)	A
YKR026C	17	0.28	1	PF01008	No	11	2 (PF01008 PF00083)	J
YDL047W	17	0.34	1	PF00149	No	18	3 (PF04499 PF00118 +)	DT
YNL317W	17	0.36	6	PF00400	5 (PF00400)	21	4 (PF00118)	A
YPL178W	17	0.25	1	PF00076	No	90	5 (PF00076)	A
YPL248C	17	0.20	9	PF03902, PF04082, PF00172	No	80	2 (PF00004)	X

YPR178W	17	0.18	8	PF00400	7 (PF00400)	109	8 (PF01423)	A
YER110C	16	0.26	13	PF02985, PF03810	7 (PF02985)	15	2 (PF04096)	YU
YAL021C	16	0.31	7	PF00560, PF03372	2 (PF00560)	72	2 (PF04153 PF00069)	K
YDL132W	16	0.34	2	PF00888	No	35	6 (PF00646)	D
YNR052C	16	0.27	2	PF04857	No	25	3 (PF00069)	A
YBR253W	16	0.27	1	PfamB/OP	No	15	1 (PF00134)	
YBR114W	16	0.05	5	PF00097, PF00271, PF00176	No	121	1 (PF00350)	L
YDR335W	16	0.23	1	PfamB/OP	No	45	3 (PF04096)	YU
YPL235W	16	0.23	4	PF06068	No	25	2 (PF00505)	K
YKR048C	16	0.07	1	PF00956	No	86	4 (PF00069)	BD
YAL029C	16	0.07	8	PF01843, PF00063, PF00612, PF02736	3 (PF00612)	39	2 (PF02985 PF00036)	Z
YLR229C	16	0.22	1	PF00071	No	12	5 (PF00786)	R
YMR139W	16	0.10	1	PF00069	No	36	2 (PF00063 PF01021 +)	G
YOR039W	16	0.14	1	PF01214	No	37	2 (PF01399 PF00076 +)	TDK
YGL130W	15	0.23	2	PF01331, PF03919	No	51	2 (PF00012)	A
YGL190C	15	0.27	7	PF00400	5 (PF00400)	64	5 (PF00118)	T
YOL090W	15	0.28	4	PF00488, PF05188, PF01624, PF05192	No	19	2 (PF01624 PF02518 +)	L
YER171W	15	0.31	6	PF06733, PF06777	No	52	2 (PF00153 PF02985)	KL
YGL161C	15	0.33	2	PF04893	No	40	10 (PF00071)	S
YGL092W	15	0.32	9	PF04096	No	450	2 (PF02985 PF03810 +)	YU
YGR092W	15	0.32	3	PF00433, PF00069	No	175	3 (PF00069)	R
YKR002W	15	0.31	2	PF04926, PF04928	No	40	1 (PF03178)	A
YLR362W	15	0.30	4	PF07647, PF00069	No	216	4 (PF00069)	T
YOR212W	15	0.26	7	PF00400	7 (PF00400)	6	5 (PF00118)	R
YJR064W	15	0.20	1	PF00118	No	30	10 (PF00400)	O
YBR126C	15	0.27	1	PF00982	No	18	3 (PF02358 PF00982)	G
YGL198W	15	0.27	1	PF04893	No	33	10 (PF00071)	S
YBR079C	15	0.32	6	PF01399	No	83	2 (PF00076)	J

YBR200W	14	0.19	4	PF00018, PF00787, PF00564	2 (PF00018)	76	2 (PF07647 PF00018 +)	C
YPL140C	14	0.29	3	PF00069	No	210	2 (PF00180 PF00069)	T
YHR041C	14	0.16	2	PfamB/OP	No	16	2 (PF00400)	
YKR001C	14	0.23	3	PF02212, PF01031, PF00350	No	79	4 (PF00176 PF00271)	UR
YMR061W	14	0.31	4	PF05843	No	46	3 (PF00076)	A
YNL263C	14	0.35	1	PF03878	No	50	11 (PF00071)	S
YJL014W	14	0.22	1	PF00118	No	6	12 (PF00400)	O
YGL115W	14	0.25	4	PF00571	4 (PF00571)	18	8 (PF00069)	C
YCR077C	14	0.35	5	PfamB/OP	No	249	8 (PF01423)	S
YER081W	13	0.14	3	PF02826, PF01842, PF00389	No	27	2 (PF00107 PF02826 +)	E
YHR061C	13	0.27	3	PF00786	No	52	2 (PF00786)	
YLR191W	13	0.32	3	PF00018, PF04088	No	71	2 (PF04757)	U
YMR001C	13	0.31	4	PF00659, PF00069	2 (PF00659)	164	2 (PF06470 PF02463 +)	D
YPL151C	13	0.22	8	PF00400	7 (PF00400)	32	2 (PF01423 PF00118)	A
YDL002C	13	0.20	1	PF00505	No	56	3 (PF00176 PF00271)	R
YMR012W	13	0.26	8	PfamB/OP	No	58	1 (PF06733)	R
YAL043C	13	0.39	6	PfamB/OP	No	80	2 (PF00400)	A
YBR103W	13	0.22	7	PF00400	5 (PF00400)	2	1 (PF02146)	B
YDR443C	13	0.17	14	PfamB/OP	No	66	1 (PF00560)	
YJR093C	13	0.17	2	PF05182	No	29	1 (PF03178)	A
YNL244C	13	0.16	1	PF01253	No	4	3 (PF01399)	J
YBL105C	13	0.21	10	PF02185, PF00433, PF00168, PF00069	3 (PF00130)	290	2 (PF00069)	T
YBR135W	13	0.13	1	PF01111	No	3	7 (PF00134)	D
YDL185W	13	0.31	6	PF02874, PF00306, PF05203	No	17	2 (PF01496)	C
YLR275W	13	0.20	1	PF01423	No	24	9 (PF01423)	A
YNL025C	13	0.26	2	PF00134	No	28	4 (PF00069)	K
YHR086W	13	0.09	4	PF00076	3 (PF00076)	126	3 (PF00076)	R
YKL166C	13	0.05	1	PF00069	No	86	4 (PF00069)	T

YMR186W	13	0.29	2	PF00183, PF02518	No	33	4 (PF00515)	O
YDL017W	13	0.22	1	PF00069	No	27	3 (PF02776 PF00205)	L
YHR027C	13	0.29	12	PF01851	6 (PF01851)	43	5 (PF00069)	O
YLR117C	13	0.18	10	PF02184	7 (PF02184)	19	2 (PF00270 PF00271 +)	D
YDR228C	13	0.34	5	PfamB/OP	No	27	2 (PF00076)	A
YNL298W	13	0.29	7	PF00786, PF00169, PF00069	No	160	2 (PF00620 PF00018 +)	T
YOR308C	13	0.28	1	PF03343	No	72	4 (PF01423)	A
YCR009C	13	0.31	1	PF03114	No	21	2 (PF04554)	U
YDR309C	13	0.23	3	PF00786	No	54	2 (PF00786)	
YDL160C	12	0.11	3	PF00270, PF00271	No	83	3 (PF01423)	A
YLR180W	12	0.32	3	PF02772, PF02773, PF00438	No	2	1 (PF01502)	H
YDR311W	12	0.17	6	PF03909	2 (PF03909)	38	1 (PF00096)	KL
YEL051W	12	0.35	1	PF01813	No	44	2 (PF01496 PF01008)	C
YNL271C	12	0.27	9	PF02181, PF06367, PF06371	No	163	2 (PF00018 PF00071 +)	TZ
YDL042C	12	0.23	2	PF04574, PF02146	No	61	1 (PF00332)	BK
YDR172W	12	0.21	7	PF03143, PF03144, PF00009	No	256	3 (PF00076)	J
YPR072W	12	0.20	4	PF04065, PF04153	No	137	2 (PF04153)	K
YDL043C	12	0.30	2	PF06220	No	47	3 (PF00076 PF00400)	A
YOL123W	12	0.27	5	PF00076	2 (PF00076)	195	2 (PF04096)	A
YBR254C	12	0.24	1	PF04628	No	11	3 (PF04051)	U
YFR034C	12	0.16	2	PF00010	No	129	3 (PF00004)	
YGR074W	12	0.34	1	PF01423	No	38	2 (PF01423)	A
YBR175W	12	0.35	5	PF00400	5 (PF00400)	0	2 (PF00270 PF00271 +)	R
YDL165W	12	0.36	1	PF04153	No	9	1 (PF00569)	KDR
YKL095W	12	0.18	1	PF04502	No	41	2 (PF00118 PF00400)	S
YGL116W	12	0.21	9	PF00400	5 (PF00400)	239	4 (PF00118)	DO
YPL256C	12	0.23	3	PF00134	No	37	2 (PF00023 PF00069)	D
YBR217W	12	0.23	1	PF04110	No	44	3 (PF00004)	O

YDR227W	12	0.32	8	PfamB/OP	No	48	2 (PF00125)	
YLR447C	12	0.20	1	PF01992	No	5	3 (PF00069 PF00106)	C
YDR378C	12	0.35	1	PF01423	No	16	9 (PF01423)	A
YFL038C	12	0.29	1	PF00071	No	36	3 (PF04893)	TU
YOL149W	12	0.34	1	PF06058	No	35	7 (PF01423)	KA
YFR021W	11	0.19	4	PfamB/OP	No	15	1 (PF00010)	S
YFR051C	11	0.35	3	PF00928, PF01217	No	122	2 (PF04053 PF01602 +)	U
YOR361C	11	0.30	5	PF00076	No	44	2 (PF01399 PF00400)	J
YBL002W	11	0.24	1	PF00125	No	42	5 (PF00176 PF00271)	B
YDR142C	11	0.38	6	PF00400	6 (PF00400)	4	4 (PF00118)	U
YGR172C	11	0.15	1	PF04893	No	57	2 (PF00071 PF04893)	U
YHR152W	11	0.22	2	PF05032	No	42	1 (PF01063)	X
YLR342W	11	0.27	9	PF02364	No	145	1 (PF04051)	M
YLR442C	11	0.19	7	PF01426	No	270	4 (PF00125)	L
YOR332W	11	0.23	1	PF01991	No	22	2 (PF01496 PF00069)	C
YBL023C	11	0.39	4	PF00493	No	70	2 (PF00493)	L
YDR523C	11	0.16	3	PF00069	No	142	2 (PF00149)	T
YGL003C	11	0.19	9	PF00400	5 (PF00400)	201	3 (PF00069)	DO
YMR273C	11	0.21	1	PfamB/OP	No	158	2 (PF00786 PF02985)	X
YOL133W	11	0.32	1	PF00097	No	40	4 (PF00888)	O
YBR274W	11	0.40	3	PF00069	No	88	1 (PF00096)	D
YIL094C	11	0.21	1	PF00180	No	17	2 (PF00069)	E
YKL104C	11	0.17	4	PF01380, PF00310	2 (PF01380)	0	3 (PF00069)	M
YMR304W	11	0.12	7	PF00443, PF00917	No	39	4 (PF00069)	O
YAL005C	11	0.25	1	PF00012	No	47	2 (PF03810 PF00226 +)	O
YDR195W	11	0.27	5	PfamB/OP	No	62	2 (PF00400)	
YBL016W	11	0.36	1	PF00069	No	2	2 (PF00069)	T
YDL116W	11	0.26	1	PF04121	No	18	3 (PF04096)	YU

YDR507C	11	0.24	7	PF05672, PF00069	No	226	5 (PF00735)	D
YFR037C	11	0.28	5	PF00569, PF04433, PF00249	No	72	3 (PF00176 PF00271)	B
YHL007C	11	0.33	7	PF00786, PF00069	No	206	3 (PF00018)	T
YJL187C	11	0.18	5	PF00069	No	364	2 (PF00564 PF00400 +)	D
YOL108C	11	0.10	1	PfamB/OP	No	41	5 (PF00010)	
YBR089C-A	11	0.03	1	PF00505	No	32	4 (PF00176 PF00271)	
YEL009C	11	0.06	2	PfamB/OP	No	43	1 (PF00569)	K
YPR119W	11	0.20	3	PF00134, PF02984	No	211	2 (PF00400 PF00069)	D
YML010W	10	0.28	13	PF03439, PF00467	5 (PF00467)	236	1 (PF02020)	K
YAL041W	10	0.25	6	PF06395, PF00621, PF00564	No	59	2 (PF00069 PF00071)	T
YBR011C	10	0.28	1	PF00719	No	21	3 (PF00069)	C
YDL101C	10	0.16	2	PF00498, PF00069	No	92	2 (PF00069)	D
YJL081C	10	0.31	1	PF00022	No	12	4 (PF00125)	Z
YLL021W	10	0.32	8	PfamB/OP	No	630	5 (PF00069)	X
YMR201C	10	0.10	4	PF05181, PF01286	No	37	1 (PF06733)	L
YDL192W	10	0.28	1	PF00025	No	3	1 (PF00025)	U
YER172C	10	0.22	13	PF00270, PF00271, PF02889	No	100	3 (PF01423)	A
YGR252W	10	0.31	4	PF00439, PF00583	No	73	1 (PF00096)	BK
YLR423C	10	0.11	1	PF04108	No	16	3 (PF00071)	X
YNL093W	10	0.32	1	PF00071	No	40	3 (PF00069)	U
YNL243W	10	0.27	6	PF07651, PF01608	No	80	2 (PF00018)	Z
YCR084C	10	0.19	10	PF00400	7 (PF00400)	97	2 (PF00125)	R
YDR404C	10	0.17	2	PF00575, PF03876	No	0	1 (PF01193)	K
YGR104C	10	0.28	3	PfamB/OP	No	28	1 (PF00134)	
YJL194W	10	0.21	3	PF00004	No	67	2 (PF00004)	LD
YER125W	10	0.24	6	PF00168, PF00397, PF00632	3 (PF00397)	111	2 (PF04425 PF02752 +)	O
YJL095W	10	0.31	6	PF00069	No	190	4 (PF00069)	T
YML065W	10	0.29	7	PF00004, PF01426	No	148	1 (PF05460)	L

YDR216W	10	0.03	13	PF00096	2 (PF00096)	145	2 (PF00400)	R
YDR416W	10	0.19	7	PfamB/OP	No	18	1 (PF06246)	A
YJR066W	10	0.19	20	PF00454, PF02260, PF02259, PF02985	4 (PF02985)	43	4 (PF00320)	L
YKR036C	10	0.32	8	PF00400	5 (PF00400)	42	5 (PF00118)	R
YLL026W	10	0.11	9	PF07724, PF00004, PF02861	2 (PF02861)	36	3 (PF00515)	O
YLR096W	10	0.26	6	PF02149, PF00069	No	111	2 (PF00018 PF07646 +)	T
YNL006W	10	0.24	6	PF00400	6 (PF00400)	0	3 (PF02985)	R
YOR106W	10	0.28	2	PF00804, PF05739	No	127	2 (PF00957 PF01602)	U
YAR007C	10	0.32	4	PF01336, PF04057	No	76	2 (PF00270)	L
YGL207W	10	0.29	6	PF00557	No	84	3 (PF00069)	E
YNL288W	10	0.30	2	PF04078	No	52	3 (PF04153)	R
YBR279W	10	0.31	1	PF03985	No	84	1 (PF05000)	K
YPL129W	10	0.32	2	PF03366	No	40	1 (PF00096)	K
YDR240C	9	0.26	4	PfamB/OP	No	77	4 (PF00076)	
YGL240W	9	0.39	1	PF03256	No	3	3 (PF00515)	DO
YLR310C	9	0.14	10	PF00618, PF00018, PF00617	No	129	3 (PF00012)	T
YNL161W	9	0.36	5	PF00433, PF00069	No	96	2 (PF03637)	R
YOR061W	9	0.29	1	PF00069	No	42	2 (PF01214 PF00069)	TDK
YPL161C	9	0.33	6	PfamB/OP	No	28	5 (PF00071)	
YPL181W	9	0.25	2	PF00628	No	347	2 (PB098478)	R
YAL032C	9	0.16	2	PF02731	No	68	2 (PF00400)	AB
YDR432W	9	0.08	4	PF00076	2 (PF00076)	118	5 (PF00076)	A
YBL093C	9	0.22	2	PfamB/OP	No	47	2 (PF06017 PF00063 +)	X
YHR023W	9	0.26	9	PF00063, PF00038	No	296	2 (PF00063 PF01843 +)	Z
YMR043W	9	0.18	3	PF00319	No	41	1 (PF00498)	K
YLR044C	9	0.15	5	PF00205, PF02776	No	6	2 (PF00176 PF00271)	EH
YMR054W	9	0.30	1	PF01496	No	60	2 (PF02874 PF00306)	C
YJL115W	9	0.23	2	PF04729	No	54	2 (PF00268)	KB

YLR015W	9	0.21	3	PfamB/OP	No	31	3 (PF00400)	BK
YIL046W	9	0.16	9	PF00646, PF00400	7 (PF00400)	142	2 (PF00240)	R
YNL127W	9	0.26	7	PF07923	No	35	1 (PF01426)	S
YNR017W	9	0.22	1	PF02466	No	47	4 (PF02953)	U
YDR238C	9	0.36	3	PF01602, PF07718	No	38	2 (PF04053 PF00400)	U
YDR468C	9	0.32	2	PF05739	No	83	1 (PF02071)	U
YIL038C	9	0.29	5	PF04065, PF04153	No	393	1 (PF02776)	K
YKR068C	9	0.24	1	PF04051	No	19	2 (PF04051 PF04099)	U
YLR288C	9	0.29	4	PfamB/OP	No	27	2 (PF00153)	
YLR418C	9	0.30	2	PF05179	No	33	1 (PF00382)	K
YBR119W	9	0.26	3	PF00076	No	82	2 (PF00076)	R
YBR289W	9	0.32	6	PF04855	No	37	1 (PF07569)	BK
YGR009C	9	0.24	5	PF05739	No	139	3 (PF00957)	U
YHR099W	9	0.18	15	PF00454, PF02260, PF02259	No	52	2 (PF00439)	TBLD
YKL139W	9	0.13	2	PF00069	No	181	3 (PF00076)	D
YLR319C	9	0.23	3	PF03915	No	129	2 (PF02181 PF06371 +)	
YMR199W	9	0.28	3	PF00134	No	45	2 (PF00069)	D
YOR089C	9	0.19	1	PF00071	No	38	3 (PF00071)	U
YER120W	8	0.23	2	PF00635	No	93	3 (PF00169)	U
YGL100W	8	0.26	5	PF00400	5 (PF00400)	6	3 (PF00400)	YU
YJR090C	8	0.37	11	PF00646	No	51	2 (PF00134 PF02776 +)	R
YOR290C	8	0.28	14	PF00439, PF00271, PF00176, PF02178	2 (PF02178)	169	2 (PF00249 PF04433)	BK
YBR221C	8	0.27	2	PF02780, PF02779	No	15	3 (PF00364)	C
YER031C	8	0.39	1	PF00071	No	45	3 (PF04893)	U
YJL061W	8	0.37	6	PfamB/OP	No	41	2 (PF03177)	
YJL141C	8	0.24	5	PF00069	No	263	2 (PF00350 PF05652 +)	R
YKL081W	8	0.29	3	PF00647, PF00043, PF02798	No	48	3 (PF00400)	J
YML001W	8	0.34	1	PF00071	No	35	2 (PF04893)	R

YIL112W	8	0.29	10	PF00023	4 (PF00023)	221	1 (PF02146)	R
YJR042W	8	0.39	1	PF07575	No	17	2 (PF00400 PF04096)	YU
YPL082C	8	0.21	17	PF00271, PF02985, PF00176	6 (PF02985)	114	1 (PF00350)	K
YBR193C	8	0.18	1	PfamB/OP	No	20	1 (PF00627)	X
YCR033W	8	0.22	11	PF03990, PF00249	2 (PF00249)	52	1 (PF02146)	K
YDR283C	8	0.17	11	PF05773, PF00069	No	101	2 (PF02518 PF00183 +)	J
YDR473C	8	0.21	4	PfamB/OP	No	41	4 (PF01423)	A
YER173W	8	0.25	1	PF03215	No	64	2 (PF00004)	DL
YKL203C	8	0.20	20	PF00454, PF02260, PF02259, PF02985	4 (PF02985)	46	1 (PF00618)	L
YGR134W	8	0.20	11	PfamB/OP	No	38	2 (PF04153 PF04065)	
YJL164C	8	0.29	1	PF00069	No	85	3 (PF00069)	T
YOR244W	8	0.19	3	PF01853	No	86	2 (PF00022)	B
YOR304W	8	0.24	6	PF00271, PF00176	No	87	2 (PF00505 PF00176 +)	K
YPL004C	8	0.35	3	PfamB/OP	No	28	4 (PF00069)	X
YPR054W	8	0.26	1	PF00069	No	35	2 (PF00069)	T
YDL005C	8	0.20	4	PfamB/OP	No	93	1 (PF01399)	
YDR155C	8	0.13	1	PF00160	No	15	1 (PF02146)	O
YER155C	8	0.24	18	PF00618, PF00620, PF00169, PF00617	No	192	1 (PF00448)	T
YML085C	8	0.28	2	PF00091, PF03953	No	17	3 (PF01302)	Z
YNL135C	8	0.21	1	PF00254	No	1	1 (PF00096)	O
YAL016W	8	0.30	12	PF02985	12 (PF02985)	15	2 (PF00149)	T
YBL106C	8	0.16	2	PfamB/OP	No	48	1 (PF02809)	U
YDL126C	8	0.34	6	PF00004, PF02359	2 (PF00004)	44	1 (PF00312)	O
YGR086C	8	0.29	1	PfamB/OP	No	64	5 (PF00069)	X
YMR236W	8	0.07	1	PF02291	No	33	1 (PF00569)	K
YOL086C	8	0.15	1	PF00107	No	1	3 (PF00176 PF00271)	Q
YOR076C	8	0.33	6	PF00009	No	129	2 (PF00270)	J
YDR207C	8	0.34	8	PF00172	No	130	2 (PF00069)	

YLR337C	8	0.40	5	PF02205	2 (PF02205)	386	4 (PF00018)	Z
YML007W	8	0.29	6	PF00170	No	143	2 (PF02985 PF03810)	X
YPR107C	8	0.40	4	PF00642	4 (PF00642)	39	2 (PF00400)	A
YER068W	8	0.36	7	PF00097, PF00076, PF00642	No	155	3 (PF04153)	K
YFR028C	8	0.29	5	PF00782	No	61	1 (PF02146)	V
YHR158C	8	0.33	11	PF01344, PF07646, PF07195	4 (PF01344)	190	3 (PF00069)	R
YJL128C	8	0.30	2	PF00069	No	297	4 (PF00069)	T
YOL038W	8	0.30	1	PF00227	No	19	3 (PF00227)	O
YOL068C	8	0.22	2	PF04574, PF02146	No	37	1 (PF00023)	BK
YOL148C	8	0.32	5	PfamB/OP	No	87	1 (PF00569)	BK
YPL218W	8	0.33	1	PF00025	No	2	1 (PF00957)	U
PH	k	aPCC	Doms	Domains	Rep.doms	Diso	Int.dom	Func.
YCR057C	51	0.67	13	PF00400, PF04047	7 (PF00400)	72	10 (PF00400)	A
YMR047C	41	0.35	10	PF04096	No	951	6 (PF03810)	YU
YDR394W	36	0.43	3	PF00004	No	23	5 (PF01399 PF00004)	O
YFL039C	34	0.36	1	PF00022	No	5	3 (PF00018 PF00307 +)	Z
YPL043W	33	0.69	5	PF00076	4 (PF00076)	53	4 (PF00270 PF00271)	A
YER012W	32	0.66	1	PF00227	No	3	12 (PF00227)	O
YDL213C	31	0.59	1	PF00076	No	77	6 (PF00270 PF00271)	
YKL145W	30	0.46	4	PF00004	No	41	6 (PF00004)	O
YKL068W	30	0.38	8	PF04096	No	756	5 (PF03810)	YU
YPR110C	28	0.45	3	PF01000	No	2	3 (PF02786 PF00289 +)	K
YCL059C	28	0.76	3	PF00013	No	36	9 (PF00400)	JD
YMR049C	27	0.67	8	PF00400	5 (PF00400)	151	4 (PF00270 PF00271)	J
YDL147W	26	0.53	4	PF01399	No	13	6 (PF00227 PF00004)	O
YDL029W	26	0.40	1	PF00022	No	10	4 (PF00069)	Z
YHR200W	25	0.65	2	PfamB/OP	No	34	10 (PF00227)	O
YGR119C	25	0.36	4	PfamB/OP	No	291	6 (PF03810)	YU

YLR293C	23	0.51	1	PF00071	No	51	5 (PF03810)	U
YBL007C	23	0.40	11	PF03983, PF00018	3 (PF00018)	470	5 (PF00018)	X
YER082C	22	0.69	6	PF00400	2 (PF00400)	98	8 (PF00400)	A
YPR016C	22	0.52	1	PF01912	No	5	3 (PF00270 PF00271 +)	J
YGL048C	22	0.44	5	PF00004	No	46	4 (PF00004)	O
YNL110C	21	0.77	1	PF00076	No	92	3 (PF00270 PF00271)	R
YMR109W	21	0.39	4	PF00018, PF00063, PF06017	No	154	6 (PF00018)	Z
YGR090W	20	0.61	3	PF03813	No	66	7 (PF00400)	S
YGR162W	20	0.43	5	PF02854	No	359	7 (PF00076)	J
YMR290C	19	0.58	4	PF00270, PF00271	No	39	4 (PF00076)	A
YGL011C	19	0.48	1	PF00227	No	16	10 (PF00227)	O
YLR074C	19	0.63	1	PF06220	No	18	2 (PF01926 PF00076)	A
YDL097C	19	0.58	2	PF01399	No	59	6 (PF00004)	O
YDR427W	19	0.44	2	PF01399	No	11	2 (PF00627 PF01399 +)	O
YCR088W	19	0.47	4	PF00018, PF00241	No	408	4 (PF00018 PF00069)	Z
YKL129C	19	0.40	4	PF00018, PF00063, PF06017	No	200	4 (PF00018)	Z
YJR121W	18	0.54	3	PF02874, PF00306, PF00006	No	32	1 (PF04911)	C
YPR041W	18	0.49	3	PF01873, PF02020	No	74	2 (PF01399 PF02854)	J
YFR052W	18	0.60	2	PF04653	No	2	6 (PF00004)	O
YLR026C	18	0.42	2	PF00804, PF05739	No	147	4 (PF04810 PF00626 +)	U
YOR116C	18	0.43	5	PF04998, PF04983, PF00623, PF04997	No	30	2 (PF01096 PF02150)	K
YBL099W	18	0.54	3	PF02874, PF00306, PF00006	No	15	1 (PF04911)	C
YDR060W	17	0.69	7	PF03914	No	102	3 (PF00270 PF00271 +)	JK
YER112W	17	0.45	2	PF01423	No	55	10 (PF01423)	A
YGR103W	17	0.72	4	PF06732, PF00533	No	78	3 (PF00076 PF00400)	A
YBR245C	17	0.42	6	PF00271, PF00176	No	71	3 (PF00176 PF00271)	K
YLR335W	17	0.48	5	PF00638	No	478	2 (PF00012 PF04096)	YU
YLR078C	17	0.59	1	PF05008	No	48	2 (PF00025 PF04810 +)	U

YNL061W	16	0.64	2	PF01189	No	98	2 (PF01926 PF00076 +)	A
YML092C	16	0.40	1	PF00227	No	5	8 (PF00227)	O
YNL132W	16	0.57	9	PF05127	No	35	4 (PF00400)	R
YLR175W	16	0.61	3	PF01509, PF01472	No	53	2 (PF00270 PF00271 +)	J
YER146W	16	0.43	1	PF01423	No	9	9 (PF01423)	A
YMR116C	16	0.56	7	PF00400	7 (PF00400)	7	2 (PF00013)	T
YBR017C	16	0.38	10	PF02985	5 (PF02985)	14	3 (PF00153 PF02985)	YU
YHR107C	16	0.37	1	PF00735	No	61	4 (PF00735)	DZU
YMR080C	15	0.44	7	PF00270	No	21	3 (PF00076)	A
YDR301W	15	0.46	8	PF03178	No	23	2 (PF00400)	A
YBR142W	15	0.63	6	PF00270, PF00271	No	125	2 (PF00270 PF00271 +)	A
YPR165W	15	0.40	1	PF00071	No	22	3 (PF00620)	R
YOR117W	15	0.48	3	PF00004	No	61	3 (PF00004)	O
YDL208W	15	0.53	1	PF01248	No	36	3 (PF00400)	A
YOR259C	15	0.45	4	PF00004	No	49	4 (PF00004)	O
YDR280W	14	0.51	2	PF03725, PF01138	No	21	3 (PF01138 PF03725)	J
YHL030W	14	0.38	19	PF02985	7 (PF02985)	42	2 (PF01399 PF01398)	S
YOL021C	14	0.58	5	PF00773	No	38	6 (PF01138)	J
YOR261C	14	0.52	2	PF01398	No	45	4 (PF00227)	O
YPR181C	14	0.45	5	PF04810, PF04811, PF04815, PF00626	No	22	3 (PF04810 PF00626 +)	U
YBR143C	14	0.40	3	PF03465, PF03464, PF03463	No	13	3 (PF02985)	J
YFR004W	14	0.55	2	PF01398	No	55	4 (PF00004)	O
YDR225W	14	0.36	1	PF00125	No	29	3 (PF00125)	B
YHR016C	14	0.38	4	PF00018, PF04366	No	169	3 (PF00018)	S
YHR166C	14	0.77	9	PF00515, PF04049	6 (PF00515)	28	2 (PF07719 PF00515)	DO
YNL287W	14	0.42	4	PF01602	No	78	2 (PF04053 PF00400)	U
YKL018W	14	0.54	5	PF00400	No	1	3 (PF00400)	ABO
YLR208W	14	0.42	6	PF00400	6 (PF00400)	5	3 (PF00400)	U

YBR010W	13	0.44	1	PF00125	No	46	3 (PF00125 PF00069)	B
YFR050C	13	0.52	1	PF00227	No	23	4 (PF00227)	O
YIL021W	13	0.40	2	PF01000	No	54	2 (PF01193)	K
YJL002C	13	0.64	1	PF04597	No	23	3 (PF00400)	O
YLR262C	13	0.38	1	PF00071	No	41	1 (PF02414)	S
YNL094W	13	0.45	3	PfamB/OP	No	57	6 (PF00018)	X
YDR097C	13	0.44	5	PF00488, PF05188, PF01624, PF05192	No	309	2 (PF01624 PF01336 +)	L
YBL038W	13	0.39	1	PF00252	No	15	2 (PF00169)	J
YOR341W	12	0.59	5	PF04998, PF04983, PF00623, PF04997	No	90	1 (PF01193)	K
YKL022C	12	0.82	11	PF00515, PF07719	6 (PF00515)	39	2 (PF00515 PF00400)	DO
YOR362C	12	0.40	1	PF00227	No	46	4 (PF00227)	O
YDR103W	12	0.47	8	PF00097	No	62	3 (PF00069)	X
YDL014W	12	0.60	1	PF01269	No	50	2 (PF00076 PF01798)	A
YJL074C	12	0.59	9	PF06470, PF02483, PF02463	No	86	3 (PF06470 PF02463 +)	D
YMR314W	12	0.59	1	PF00227	No	15	4 (PF00227)	O
YGR245C	12	0.75	3	PF05285	No	102	3 (PF01926)	DZ
YER006W	12	0.68	4	PF01926	No	57	3 (PF00076)	R
YER126C	12	0.85	1	PF01201	No	35	2 (PF01926 PF00400)	R
YDL007W	12	0.63	5	PF00004	No	44	2 (PF01399 PF00004)	O
YLR127C	12	0.63	6	PF00888	No	38	3 (PF00515)	DO
YLR277C	12	0.57	5	PF07521, PF00753	No	28	2 (PF00400)	A
YBR088C	12	0.44	2	PF00705, PF02747	No	13	5 (PF00004)	L
YHR089C	12	0.62	1	PF04410	No	84	2 (PF00076)	J
YJL109C	12	0.66	6	PfamB/OP	No	30	4 (PF00400)	S
YOR270C	11	0.60	1	PF01496	No	50	2 (PF02874 PF00306)	C
YDR101C	11	0.61	3	PF00557	No	24	1 (PF05285)	R
YAR002W	11	0.47	1	PfamB/OP	No	147	2 (PF00012 PF02985 +)	Y
YFR002W	11	0.38	3	PF04097	No	39	1 (PF07926)	D

YHR052W	11	0.71	1	PfamB/OP	No	35	3 (PF00076 PF00400)	S
YOR272W	11	0.63	6	PF00400	6 (PF00400)	5	3 (PF00076)	Z
YNR003C	11	0.63	1	PF05158	No	20	1 (PF00382)	K
YNR053C	11	0.49	3	PF01926	No	27	2 (PF01423)	R
YBR084W	11	0.60	3	PF02882, PF00763, PF01268	No	13	1 (PF05672)	H
YLR384C	11	0.40	3	PF04762	No	53	1 (PF04055)	K
YDL065C	11	0.43	1	PF04614	No	62	3 (PF04757)	U
YDR496C	11	0.55	7	PF00806	4 (PF00806)	53	2 (PF00076)	J
YJR076C	11	0.47	2	PF00735	No	92	5 (PF00735)	DZU
YLR196W	11	0.40	7	PF00400	3 (PF00400)	31	5 (PF00118)	S
YNL016W	11	0.39	4	PF00076	3 (PF00076)	77	5 (PF00076)	R
YER157W	11	0.49	3	PF04136	No	33	2 (PF00957)	U
YMR197C	11	0.47	1	PF05008	No	29	6 (PF05739)	U
YOL139C	11	0.55	1	PF01652	No	32	4 (PF00076)	J
YBL050W	10	0.45	5	PF02071	3 (PF02071)	14	6 (PF05739)	U
YGR120C	10	0.44	1	PfamB/OP	No	10	1 (PF06419)	
YPR010C	10	0.45	10	PF06883, PF00562, PF04561, PF04567	No	16	1 (PF02260)	K
YLL011W	10	0.63	7	PF04158, PF00400	6 (PF00400)	54	5 (PF00118)	A
YOL041C	10	0.50	4	PF00076	No	110	2 (PF00270 PF00271 +)	R
YOR151C	10	0.48	8	PF04566, PF00562, PF04561, PF04567	No	16	1 (PF05000)	K
YNL172W	10	0.76	8	PfamB/OP	No	53	3 (PF00515)	DO
YPL012W	10	0.73	4	PfamB/OP	No	71	3 (PF00076)	S
YAR003W	10	0.57	7	PF00400	4 (PF00400)	23	2 (PF00400)	R
YJL033W	10	0.56	6	PF00270, PF00271	No	157	3 (PF00076)	A
YJR063W	10	0.50	2	PF02150, PF01096	No	11	1 (PF00177)	K
YBL084C	10	0.86	8	PF07719, PF00515, PF05462	6 (PF07719)	149	2 (PF00515)	D
YBL004W	10	0.75	11	PF07539	No	37	3 (PF00076)	V
YDL134C	10	0.47	2	PF00149	No	63	2 (PF00069)	T

YLR314C	10	0.50	3	PF00735	No	99	4 (PF00735)	DZU
YOL094C	10	0.54	2	PF00004	No	4	5 (PF00004)	L
YOL145C	10	0.49	15	PF07719, PF00515	4 (PF00515)	100	1 (PF05000)	P
YGR156W	10	0.44	3	PF00076	No	64	1 (PF03178)	A
YLL036C	10	0.32	5	PfamB/OP	No	17	1 (PF06246)	R
YBR087W	10	0.66	2	PF00004	No	19	5 (PF00004)	DL
YDL008W	10	0.88	1	PF00097	No	33	3 (PF00515)	DO
YJR068W	10	0.54	2	PF00004	No	26	4 (PF00004)	L
YHR069C	10	0.47	3	PfamB/OP	No	27	4 (PF01138 PF03725)	A
YIL109C	10	0.45	8	PF04810, PF04811, PF04815, PF00626	No	193	2 (PF05739)	U
YJL069C	10	0.83	5	PF00400	3 (PF00400)	126	5 (PF00400)	R
YOR249C	10	0.86	5	PfamB/OP	No	18	3 (PF00515)	
YLR291C	9	0.66	1	PF01008	No	12	2 (PF01008)	J
YLR222C	9	0.44	13	PF00400	10 (PF00400)	1	4 (PF00400)	A
YLR212C	9	0.41	2	PF00091, PF03953	No	22	2 (PF01920 PF02996 +)	Z
YNL002C	9	0.76	3	PF00327	No	39	2 (PF00076)	J
YNL102W	9	0.47	7	PF03104, PF00136	No	166	1 (PF04857)	L
YPL213W	9	0.40	1	PfamB/OP	No	50	4 (PF01423)	A
YPR103W	9	0.66	1	PF00227	No	17	5 (PF00227)	O
YDR224C	9	0.37	1	PF00125	No	42	3 (PF00125)	B
YDR244W	9	0.36	7	PF00515	4 (PF00515)	210	1 (PF00018)	R
YER094C	9	0.46	1	PF00227	No	2	5 (PF00227)	O
YGL004C	9	0.64	4	PF00400	2 (PF00400)	2	5 (PF00004)	R
YOR224C	9	0.59	1	PF03870	No	1	3 (PF05000 PF04997 +)	K
YDL195W	9	0.57	13	PF00400	5 (PF00400)	501	2 (PF04810 PF00626 +)	U
YGR195W	9	0.57	2	PF03725, PF01138	No	23	2 (PF01138 PF03725)	J
YIL115C	9	0.39	9	PfamB/OP	No	780	4 (PF03810)	YU
YJR045C	9	0.36	1	PF00012	No	44	2 (PF02466)	O

YJR065C	9	0.64	1	PF00022	No	20	2 (PF00018)	Z
YER136W	9	0.36	1	PF00996	No	23	9 (PF00071)	O
YFR036W	9	0.77	1	PfamB/OP	No	21	3 (PF00515)	
YOR036W	9	0.36	2	PF05739	No	109	1 (PF02071)	U
YJL157C	9	0.39	8	PF00097	No	35	3 (PF00134)	X
YLR197W	9	0.78	4	PF01798	No	33	3 (PF00400)	AJ
YPR187W	9	0.56	1	PF01192	No	17	3 (PF05000 PF04997 +)	K
YDR118W	9	0.85	6	PfamB/OP	No	17	2 (PF00515)	DO
YFL008W	9	0.52	8	PF06470, PF02483, PF02463	No	99	2 (PF06470 PF02463 +)	D
YNL118C	9	0.43	7	PF00293, PF05026	No	61	3 (PF01423)	A
YPR088C	9	0.43	4	PF02978, PF02881, PF00448	No	68	2 (PF05383)	U
YER009W	9	0.40	1	PF02136	No	1	3 (B005278)	U
YKL059C	9	0.41	4	PF04564, PF00098	No	39	2 (PF00400)	O
YGL070C	8	0.39	2	PF02150, PF01096	No	12	2 (PF05000 PF04997 +)	K
YGL200C	8	0.71	1	PF01105	No	0	2 (PF04810 PF00626 +)	U
YGR020C	8	0.41	1	PF01990	No	7	2 (PF01496)	C
YJR060W	8	0.45	3	PF00010	No	136	2 (PF07716)	K
YNL290W	8	0.49	2	PF00004	No	16	4 (PF00004)	L
YOR250C	8	0.42	2	PF06807	No	15	1 (PF03178)	A
YOR260W	8	0.82	5	PfamB/OP	No	25	3 (PF01008)	J
YBL041W	8	0.59	1	PF00227	No	7	1 (PF00096)	O
YBR102C	8	0.38	5	PfamB/OP	No	51	1 (PF02845)	U
YCR002C	8	0.57	1	PF00735	No	27	4 (PF00735)	DTZ
YEL002C	8	0.67	1	PF03345	No	17	1 (PF02109)	O
YGL153W	8	0.67	2	PF04695	No	84	1 (PF00018)	MOU
YLR113W	8	0.42	2	PF00069	No	24	4 (PF00069)	T
YNL113W	8	0.55	1	PF01193	No	46	2 (PF05000 PF01000 +)	K
YPL093W	8	0.66	3	PF06858	No	125	2 (PF00076)	R

YBR154C	8	0.43	2	PF03871, PF01191	No	18	3 (PF05000 PF04997 +)	K
YDR264C	8	0.44	8	PF00023, PF01529	4 (PF00023)	24	2 (PF00069)	R
YDL225W	8	0.49	3	PF00735	No	121	4 (PF00735)	DZU
YDR395W	8	0.43	5	PF03810	No	17	2 (PF04096)	YU
YGR135W	8	0.46	1	PF00227	No	16	3 (PF00227)	O
YKL085W	8	0.59	2	PF02866, PF00056	No	18	3 (PF00069)	C
YDR356W	8	0.41	5	PF00261, PF00038	No	70	2 (PF04130 PF00036)	
YBR127C	8	0.35	3	PF02874, PF00306, PF00006	No	24	2 (PF01496)	C
YHR077C	8	0.63	5	PF02854, PF04050	3 (PF02854)	46	1 (PF00009)	A
YOR157C	8	0.36	1	PF00227	No	18	3 (PF00227)	O
YBR118W	8	0.56	3	PF03143, PF03144, PF00009	No	19	2 (PF00043 PF02798 +)	J
YNL138W	8	0.54	1	PF01213	No	46	2 (PF00018)	ZT
YPR108W	8	0.66	4	PF07719, PF01399	No	11	3 (PF01399)	O
YER179W	8	0.45	1	PF00154	No	22	2 (PF00071 PF00176 +)	DL
YFL059W	8	0.40	1	PF01680	No	3	3 (PF01174)	H
YHR119W	8	0.49	9	PF00856, PF00076	No	168	3 (PF00400)	BK
YLR409C	8	0.56	11	PF04192, PF00400	7 (PF00400)	14	4 (PF00400)	R
